# Exosomes mediate Zika virus transmission through SMPD3 neutral Sphingomyelinase in cortical neurons

**DOI:** 10.1080/22221751.2019.1578188

**Published:** 2019-03-01

**Authors:** Wenshuo Zhou, Michael Woodson, Michael B. Sherman, Girish Neelakanta, Hameeda Sultana

**Affiliations:** a Department of Biological Sciences, Center for Molecular Medicine, Old Dominion University, Norfolk, VA, USA; b Department of Biochemistry and Molecular Biology, University of Texas Medical Branch, Galveston, TX, USA; c Sealy Center for Structural Biology and Molecular Biophysics, University of Texas Medical Branch, Galveston, TX, USA; d Department of Medicine, Division of Infectious Diseases and International Health, University of Virginia School of Medicine, Charlottesville, VA, USA

**Keywords:** Exosomes, ZIKA virus, transmission, cryo-EM, neutral Sphingomyelinase, GW4869, cortical neurons

## Abstract

The harmful effects of ZIKA virus (ZIKV) infection are reflected by severe neurological manifestations such as microcephaly in neonates and other complications associated with Guillain-Barré syndrome in adults. The transmission dynamics of ZIKV in or between neurons, or within the developing brains of the foetuses are not fully understood. Using primary cultures of murine cortical neurons, we show that ZIKV uses exosomes as mediators of viral transmission between neurons. Cryo-electron microscopy showed heterogeneous population of neuronal exosomes with a size range of 30–200 nm. Increased production of exosomes from neuronal cells was noted upon ZIKV infection. Neuronal exosomes contained both ZIKV viral RNA and protein(s) that were highly infectious to naïve cells. RNaseA and neutralizing antibodies treatment studies suggest the presence of viral RNA/proteins inside exosomes. Exosomes derived from time- and dose-dependent incubations showed increasing viral loads suggesting higher packaging and delivery of ZIKV RNA and proteins. Furthermore, we noted that ZIKV induced both activity and gene expression of neutral Sphingomyelinase (nSMase)-2/SMPD3, an important molecule that regulates production and release of exosomes. Silencing of SMPD3 in neurons resulted in reduced viral burden and transmission through exosomes. Treatment with SMPD3 specific inhibitor GW4869, significantly reduced ZIKV loads in both cortical neurons and in exosomes derived from these neuronal cells. Taken together, our results suggest that ZIKV modulates SMPD3 activity in cortical neurons for its infection and transmission through exosomes perhaps leading to severe neuronal death that may result in neurological manifestations such as microcephaly in the developing embryonic brains.

## Introduction

Mosquito-borne Zika virus (ZIKV) is a positive sense single-stranded RNA virus that belongs to the flavivirus genus of the family Flaviviridae [1]. ZIKV has been in the spotlight due to its recent epidemic outbreak in Brazil and spread in several parts of the Western Hemisphere including the United States of America [1–3]. Since more than 60 years, ZIKV existed in the Zika forest of Uganda and recently has become an International prominence and Public Health Emergency of International Concern (PHEIC) [1–3]. ZIKV belongs to the Spondweni serocomplex and is closely related to dengue (DENV) and West Nile virus (WNV) [1]. Mostly, infections with ZIKV are asymptomatic (∼80%) with flu-like symptoms and simple associated clinical manifestations. The sexual transmission of ZIKV, with replicative viral particles being detectable in semen for at least two months, proposes it to be a significant global threat and a pathogen of high priority concern to the public health [4,5]. Typically, *Aedes aegypti* mosquitoes transmit most of the ZIKV infections to humans. However, ZIKV can also be transmitted through sexual contacts and transfusions of human blood at the clinical side. In humans, vertical transmission of ZIKV from mother to neonates is of the highest concern and has been of focus due to the associated neurological manifestations [1–3,6–8]. ZIKV infection has been shown to affect both the Central Nervous system (CNS) and the Peripheral Nervous System (PNS) and is associated with severe neurological complications such as Guillain-Barré syndrome (GBS with muscle weakness and paralysis) and the attentive manifestation of microcephaly [1–3,6–12]. Microcephaly, a less studied neurodevelopmental disorder is a marked reduction in brain size and intellectual disability with defective cell proliferation and severe death of cortical progenitor cells and their neuronal progeny [6,8,11].

Although emergence of ZIKV-associated congenital microcephaly and neuropathogenesis is being studied extensively, this line of research is currently very limited. Since January 2016, significant and stunning progress has been made in developing stem cell-based cellular and animal models [11,13]. In addition to the identification of underlying molecular mechanisms and development of therapeutics and vaccines, involvement of human tissues and samples has led to the understanding of ZIKV infections [2,3,7,11,13]. In a developmental mouse model of ZIKV infection, it has been shown that astrocytes were targeted throughout the brain upon entry into the CNS after peripheral inoculations [3]. ZIKV has been shown to efficiently infect and replicate in mouse neural stem cells (mNSCs), mouse astroglial cells and different regions of brain including neocortex and hippocampal regions (CA1 and CA3), thereby raising several concerns related to long-term memory problems [3,9–12,14]. ZIKV RNA has been detected in neural tissues, human neural progenitors, matured neurons and has been correlated with an increase in the apoptosis-related genes in those neuronal cells [3,9,10,12,14]. The cerebral cortex, a four-layered structure that mediates the higher cognitive functions such as learning and memory has been severely affected in microcephalic patients [6]. Two independent studies have also shown that ZIKV infection can drastically reduce the growth of neural stem cells and brain organoids that can be directly co-related to the ZIKV-associated congenital microcephaly [8,15,16]. A comparative analysis approach in the developing neocortex has identified ZIKV specific alterations and preferential infection of neural stem cells [17]. However, this study does not address the critical steps of how ZIKV reaches the brain. Also, the transmission dynamics of ZIKV in and between neurons or neural stem cells is largely unknown.

Our recent study showed that Langat virus, a virus closely related to tick-borne encephalitis virus (TBEV) uses neuronal exosomes to transmit between cells [18]. Exosomes are small (30–250 nm) bioactive functional vesicles derived from the endo-lysosomal system that exit into the surrounding microenvironments [19–25]. Exosomes are derived from mostly all of the mammalian cells and they have been shown to contain cell and cell-state specific cargo of proteins, mRNA, and miRNA [26–31]. Recent discoveries of functional RNA and miRNA in the exosomes has increased the attention of many researchers that has led to the emergence of numerous studies in the identification of novel molecules present in the exosomes [28–30,32]. In various pathological conditions that include tumours, viral infections and tissue damage, exosomes aid in transmission of cargo from these sites to other(s) within the human body [32,33]. Exosomes have been shown to play both neuroprotective and toxic roles in the CNS [33]. Several reports have suggested neuronal exosomes as novel therapeutic targets for neurological disorders such as Alzheimer’s disease [33–37]. We have hypothesized that ZIKV uses exosomes to transmit to other neurons and spread infection through neuronal connectivity in developing embryonic foetal and neonatal brains. Our study shows that mouse cortical neuronal cell-derived exosomes carry ZIKV infectious RNA and proteins that mediate viral transmission to other neurons, thereby aiding in viral dissemination throughout the CNS. Also, our work suggests that neutral Sphingomyelinase SMPD3 (also referred as nSMase2), an enzyme sphingomyelin phosphodiesterase is involved in ZIKV infection, replication, and mediates infectious viral RNA and protein transmission via neuronal exosomes. SMPD3 hydrolyses sphingomyelin to phosphocholine and ceramide and is predominantly expressed in the neurons of the CNS to control postnatal growth and development [38–40]. In addition, SMPD3 has been shown to reside in Golgi apparatus and is ubiquitously expressed, thereby suggesting its important role in ZIKV replication [40,41]. Overall, our study not only provides a model in understanding the importance of exosomes but can also lead in the progress of new strategies that include the development of exosomal-based drugs or therapeutics and/or vaccines to interrupt the ZIKV infections in neonatal brains.

## Results

### ZIKV infects mouse cortical neurons in a time- and dose-dependent manner

**Figure 1.**
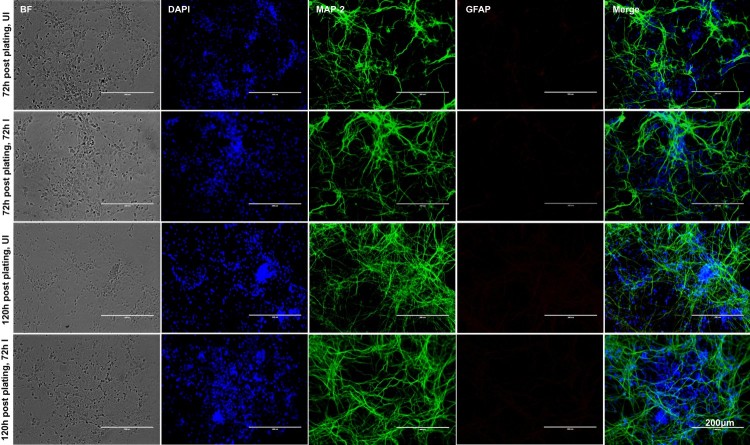
Murine cortical neurons in primary cultures are differentiated as neurons from progenitor cells. Phase contrast and fluorescent microscopic images showing primary cultures of cortical neurons from uninfected (UI) or ZIKV (I) infected (MOI 5; 72 h p.i.) groups at two different time points (72 and 120 h post plating). Cortical neurons were stained for neuronal marker (MAP-2) or astrocyte marker (GFAP) to show the presence of neurons and absence of glial cells. DAPI staining for nuclei serve as internal control. Uninfected neurons serve as control. Representative images obtained from EVOS FL system are shown. Scale bar indicates 200 μm in all panels.

**Figure 2.**
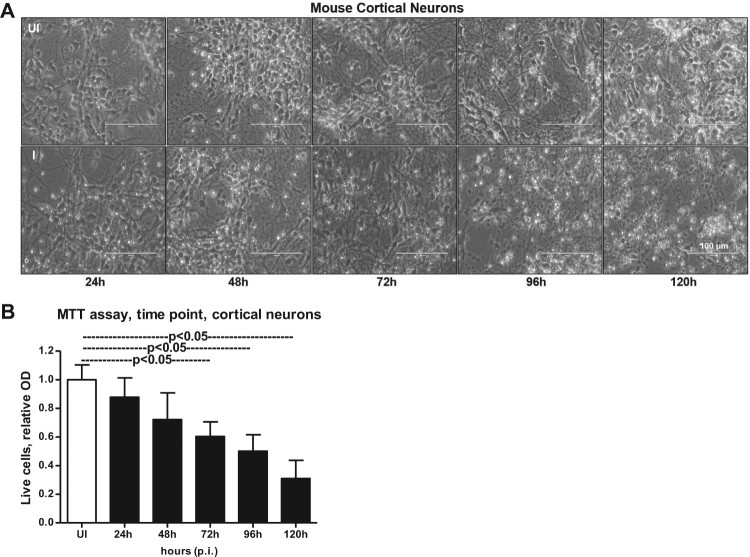
Murine cortical neurons in primary cultures are susceptible to ZIKV infection at longer incubation time. (A) Phase contrast images showing primary cultures of cortical neurons from uninfected (UI) and ZIKV (I) infected (MOI 5) groups at different time points (of 24, 48, 72, 96 and 120 h p.i.). Uninfected neurons serve as control. Representative images obtained from EVOS FL system are shown. Scale bar indicates 100 μm in all panels. (B) MTT assay showing cell viability of mouse cortical neurons at different time points (of 24, 48, 72, 96, and 120 h p.i.) upon ZIKV infection (5 MOI). Relative optical density shows live cells in culture. Uninfected controls kept for 120 h were considered as control.

Smaller head circumference, intellectual ability and seizures that characterizes microcephaly, has been strongly associated with ZIKA virus (ZIKV) infections [6,8,11,15–17,42]. Also, a correlation between ZIKV and reduced neuronal differentiation or increased cell death in neuronal cells has been shown [2,3,6,9–12,14,43]. In order to determine the correct time of neuronal differentiation and to address if cells in primary cultures are perhaps not in progenitor state during ZIKV infection, we plated neuronal cultures for either 72 or 120 h (post-plating) followed by infection with ZIKV (for another 72 h p.i., 5 MOI; Multiplication of Infection). We infected freshly isolated primary cultures of murine cortical neurons from embryonic (E16) developing mouse brains. Primary cultures were stained for both neuronal marker MAP-2 (Microtubule Associated Protein-2) or with GFAP (Glial Fibrillary Acidic protein) an intermediate filament protein indicated as astrocyte marker (Figure 1). At both post plating time points of 72 or 120 h, we found that progenitor cells were fully differentiated as neurons as revealed by the staining for MAP-2 (green) followed by detection with Alexa Fluor 488 (Figure 1). We did not find any astrocytes or other glial cells or neuronal precursor cells (NPCs) (for GFAP positive staining, and detection with Alexa Fluor 594) in our primary cultures of murine cortical neurons at 72 or 120 h of post plating, followed by infection with ZIKV (for another 72 h) (Figure 1). Also, the bright field (BF) images, predominantly showed neurons in our primary cultures at both 72 and 120 h post plating of cells (Figure 1). Infection of primary cultures of cortical neurons at/after 72 or 120 h post plating had no differences with ZIKV infection. Some of the DAPI-positive but weak MAP-2 stained cells were found to have strong staining for ZIKV Envelope (E) protein (detected by 4G2 monoclonal antibody, followed by Alexa Fluor 594 secondary antibody) (Supplementary Fig. 1). We assume that neurons perhaps have retracted their neurites due to severe ZIKV infection thereby leading to neuronal death in those weak MAP-2 stained cells. We also found that ZIKV readily infected (5 MOI) murine cortical neurons with increased cell death at 96 h post infection (p.i.) in comparison to the uninfected control (Figure 2(A)). In addition, cortical neuronal cell death with ZIKV infection was severe at 120 h p.i., in comparison to infection at early time points, suggesting longer incubation times lead to severe damage and neuronal death, perhaps resulting in tissue loss (Figure 2(A)). Several neuronal connections were eliminated and severe damage was observed due to massive neuronal cell death in murine cortical neurons at 120 h p.i., (Figure 2(A)). We did not find any morphological changes or cell loss at earlier tested time points (24, 48 and 72 h p.i.) of ZIKV infection (Figure 2(A)). The quantitative analysis for assessing cell viability of primary cultures of cortical neurons by MTT assay showed reduced number of viable cells at later time points (72, 96 and 120 h) of ZIKV infection (Figure 2(B)).

**Figure 3.**
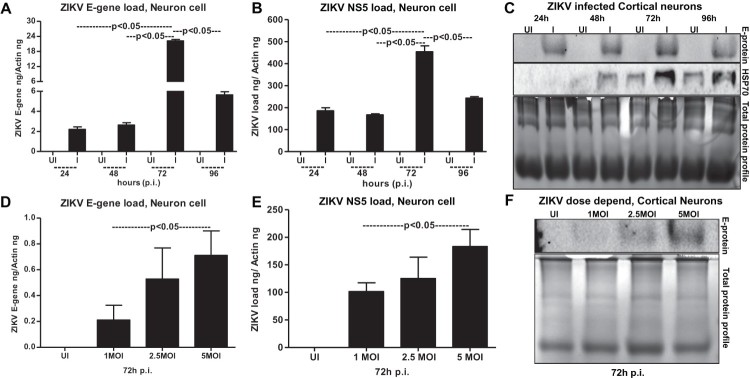
ZIKV infects primary cultures of cortical neurons in a time- and dose-dependent manner. QRT-PCR analysis showing ZIKV loads (MOI 5) determined by either E-gene (A) or NS5 (B) mRNA transcripts in neuronal cells at different time points (of 24, 48, 72 and 96 h p.i.). Uninfected cells (UI) at indicated time points served as controls. (C) Immunoblotting analysis showing ZIKV E-protein levels at different time points (24, 48, 72 and 96 h p.i. and at 5MOI). HSP70 loads serve as internal control. ZIKV loads at different MOI of infection (1, 2.5, 5 MOI) determined by either E-gene (D) or NS5 (E) mRNA transcripts is shown from 72 h p.i. (F) Immunoblot showing viral E-protein loads in cortical neurons upon infection with various doses (1, 2.5, 5 MOI). Uninfected cells were used as control in all panels. Total protein profiles shown by Coomassie-stained gels (in C and F) serve as loading controls. ZIKV loads for E-gene or NS5 mRNA transcripts are shown in both (A, D) or (B, E). E-gene or NS5 transcript levels were normalized to mouse beta-actin in (A, D) or (B, E). *P* value determined by Student’s two-tail *t*-test is shown.

To test that neuronal cell loss, is due to longer incubations with ZIKV infection, we performed an independent experiment with different doses (MOI 1, 2.5 and 5) of ZIKV. At 72 h p.i., we did not find much considerable morphological changes in cortical neurons infected with ZIKV at tested doses (of MOI 1, 2.5 and 5) (Supplementary Fig. 2A). However, quantitative analysis using MTT assay showed some reduction in cell viability at 72 h post-ZIKV infection with 5 MOI (Supplementary Fig. 2B). These data suggest that neuronal cell death at 96 h p.i., and onwards is mostly due to the result of prolonged infection of neurons with ZIKV and the neuronal stress related to infection. QRT-PCR analysis performed on the neuronal samples collected at different time points revealed that ZIKV loads (both E-gene and NS5 mRNA transcripts) were significantly (*P* < 0.05) high at 72 h p.i., in comparison to the other tested time points (of 24, 48 and 96 h p.i.,) (Figure 3(A,B)). The lower loads of ZIKV at 96 h p.i. is perhaps due to the severe neuronal cell death observed at this time point (Figure 3(A,B)). Immunoblotting analysis (using highly cross-reactive 4G2 monoclonal antibody that recognizes the viral Envelope (E)-protein) showed similar results with enhanced ZIKV E-protein at 72 h p.i., in comparison to the loads at other tested time points (Figure 3(C)). It was also observed that HSP70 (heat-shock protein 70, an enriched marker in mammalian exosomes) loads were enhanced upon ZIKV infection at 48, 72 and 96 h p.i., in comparison to their respective uninfected controls (Figure 3(C)). HSP70 levels were minimally detected at 24 h p.i. in the tested conditions (Figure 3(C)). In addition, a dose-dependent increase in ZIKV loads was evident both at the RNA (E-gene and NS5 mRNA transcripts) and protein levels when cortical neurons were infected with 1, 2.5 or 5 MOI at 72 h p.i. (Figure 3(D–F)). In both time and dose–response data (Figure 3(A,B,D,E)), NS5 mRNA amplification was found to be higher when compared with the E-gene amplification, hence we used NS5 detection in our further analysis. Total protein profile gel images served as loading controls in the immunoblotting analysis (Figure 3(C,F)). Densitometry analysis from total cell lysates showed the quantitative differences in E-protein (from both the time point and dose–response) and HSP70 levels observed between the ZIKV infected (MOI 5) and uninfected controls (Supplementary Fig. 3A–C). Collectively, these data suggest time- and dose-dependent infection kinetics of ZIKV in mouse cortical neurons.

### Cortical neuronal cell-derived exosomes contain ZIKV RNA and protein

**Figure 4.**
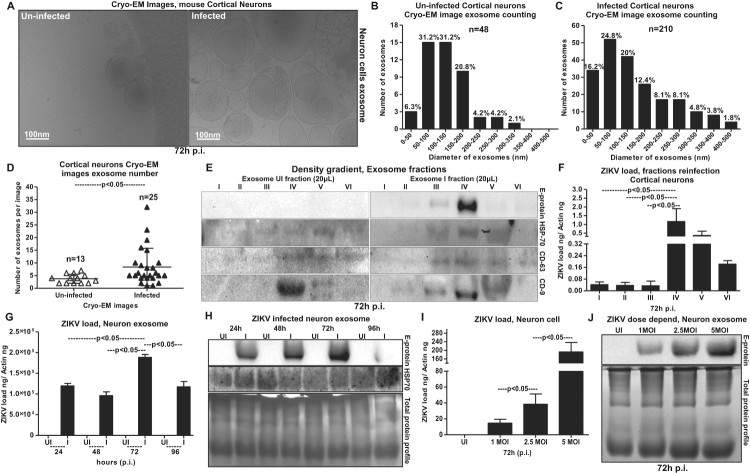
Exosomes derived from murine cortical neuronal cells are in heterogenous populations and contain infectious ZIKV RNA and E-protein. (A) Cryo-EM representative images showing exosomes isolated from uninfected (UI) or ZIKV-infected (I) (MOI 5; 72 h p.i.), cortical neuronal cells. Scale bar indicates 100 nm. Size distribution of exosomes isolated from uninfected (B) or ZIKV-infected (C) neuronal cells are shown. The Y-axis represents exosome number and X-axis indicate exosome size in diameter (e.g. 0–50 nm). N indicates total number of exosomes counted from different cryo-EM images. Percentages were calculated based on the total number of exosomes in each size range. (D) Exosome numbers counted from uninfected and ZIKV-infected groups is shown. The *Y*-axis represents exosome number and *X*-axis indicates samples analysed. *N* indicates total number of images used in this counting analysis. *P* value determined by Student’s two-tail *t*-test is shown. (E) Immunoblotting images from OptiPrep density gradient exosomes preparation (DG-Exo-isolation) showing enhanced ZIKV-E (MOI 5, 72 h p.i.) protein loads and presence of HSP70, CD63 and CD9 (exosomal markers) in different exosome fractions. Exosomes derived from uninfected cells served as control. Roman numerical indicates the fraction numbers (I being the top and VI being the small fraction at the bottom of the iodixanol density gradient). (F) Infection (72 h p.i.) of naïve primary cultures of murine cortical neurons showing increased infectivity and transmission from infectious exosomal fractions four, five and six in comparison to other lower fractions (1–3). (G) QRT-PCR showing ZIKV (I) RNA loads (MOI 5) from cortical neuronal cell-derived exosomes at different time points (24, 48, 72 and 96 h p.i.). Uninfected (UI) group from each respective time point serve as controls. (H) Immunoblotting showing the ZIKV-E protein loads (MOI 5) and HSP70 levels in neuronal cell-derived exosomes isolated at different time point samples (24, 48, 72, and 96 h p.i.). HSP70 loads show the presence and enrichment of exosomal marker and serve as internal control. (I) ZIKV loads from neuronal cell-derived exosomes collected from cortical neurons infected with different doses (MOI 1, 2.5 and 5) at 72 h p.i., is shown. (J) Immunoblotting showing ZIKV-E protein loads at different doses (MOI 1, 2.5 and 5, at 72 h p.i.) of infection (I) in neuronal cell-derived exosomes collected from infected-cortical neurons. Uninfected (UI) cell-derived exosomes collected from uninfected cortical neurons serve as control in (I) and (J). Total protein profiles serve as control in (H) and (J). In both (G) and (I), ZIKV NS5 transcripts were normalized to mouse beta-actin transcripts. *P* value determined by Student’s two-tail *t*-test is shown.

Regardless of the importance of ZIKV infection and its cause of severe neurological complications such as GBS and the more attentive manifestation of microcephaly in neonates, we know little about the transmission modes of ZIKV within the neonatal and adult brain cells. Increased HSP70 levels in neuronal cells upon infection (Figure 3(C)) suggest that ZIKV perhaps uses neuronal exosomes for transmission and spread of infection in the brain. It has been shown that cortical neurons release exosomes in culture that contain enriched exosomal markers [18,44]. Due to increased viral loads at 72 h p.i., we considered this time point for the isolation of exosomes from cortical neuronal cultures. Using either density gradient centrifugation technique; OptiPrep™ (DG-Exo isolation), or by differential ultracentrifugation, we isolated exosomes from murine cortical neurons [18,45,46]. Detailed protocols and descriptions are published in our recent work on neuronal exosomes [18]. Cryo-Electron Microscopy (EM) performed on neuronal cell-derived exosomes showed their sizes in the ranges of 30–200 nm in diameter (Figure 4(A)) that are similar to exosomes isolated from other mammalian cells. Quantitative analysis of the heterogeneous populations of neuronal exosomes was performed to determine any differences between ZIKV-infected and uninfected cell-derived exosomes (Figure 4(B,C)). We found that highest percentage of exosomes were of sizes between 50–100 nm and 100–150 nm (in diameter) in both uninfected and ZIKV-infected groups (Figure 4(B,C)). However, ZIKV infected neuronal cell-derived exosomes had increased percentages in 0–50 nm but decreased percentages in 100–150 nm and 150–200 nm sizes in comparison to the uninfected group (Figure 4(B,C)). Also, higher percentages of larger exosomes or Extracellular Vesicles (EVs) of sizes 200–500 nm were observed upon ZIKV infection in comparison to the uninfected group (Figure 4(B,C)). In addition, we noted higher number of exosomes in cryo-EM images collected from ZIKV-infected (*n* = 25) group in comparison to the uninfected (*n* = 13) group (Figure 4(D)). These data suggest increased production and or release of neuronal exosomes upon ZIKV infection.

To find whether ZIKV proteins are evident in neuronal exosomes, we collected six different fractions from samples collected at 72 h p.i., following the OptiPrep™ (DG-Exo) isolation method as described in [18,45]. Neuronal HSP70 was detected in 20 µl of the infected exosomes from three to five fractions. Barely detectable levels of HSP70 were noted in fractions one, two and six but enhanced levels were noted in fractions three to five (Figure 4(E)). HSP70 loads were also detected in exosomal fractions three-five obtained from uninfected cortical neuronal cells, but with higher levels in fractions five (Figure 4(E)). Additionally, for detailed characterization of exosomes, we detected the presence of other exosomal markers such as CD63 and CD9 in fractions from both ZIKV-infected and uninfected groups (Figure 4(E)). CD63 loads were enriched in fractions three-six in ZIKV infected and fractions four-six in uninfected controls. Barely detectable loads were observed in one and two fractions in both infected and uninfected groups (Figure 4(E)). CD9 was also enriched in fractions four and five in both ZIKV-infected and uninfected controls, and detectable levels were found in fractions two and three of the infected group (Figure 4(E)). In other fractions one and six, we could not detect CD9 loads (Figure 4(E)). We also found that ZIKV E-protein levels were enhanced in infected exosomal fraction four, however, other fractions (two, three and five) also showed weak signal for E-protein (Figure 4(E)). As expected, ZIKV E-protein was not detected in any uninfected fractions (Figure 4(E)). Since most of the ZIKV-E protein was detected in fraction four (the fraction with enhanced levels of all tested exosomal markers), we assessed this fraction for the presence of more viral RNA/proteins and their correspondence to increased infectivity on the naïve recipient cells. We found that naïve primary cultures of murine cortical neurons infected via incubations (for 72 h) with infectious exosomes from different fractions (collected from ZIKV-infected cortical neurons, at 72 h p.i., and 5 MOI) showed highest infectivity with fraction four (Figure 4(F)). Exosomes from lower fractions five, and six also showed increased infectivity when compared with the upper (1–3) fractions (Figure 4(F)). Next, we tested the loads of ZIKV in exosomes collected from cortical neurons infected at different time points (of 24, 48, 72 and 96 h p.i., 5 MOI). Similar to viral loads observed in cortical neuronal cells, ZIKV RNA loads were significantly (*P* < 0.05) higher at 72 h p.i., in comparison to the other tested time points (of 24, 48 and 96 h p.i.) (Figure 4(G)). The lower loads of ZIKV at 96 h p.i., is perhaps due to the neuronal cell death observed at this time point (Figure 2(A)). We assume that less number of exosomes were released due to massive death of neurons at 96 h p.i., (Figure 4(G)). Immunoblotting with 4G2 antibody showed increased ZIKV E-protein loads at 72 h p.i., in a time-dependent manner in comparison to the viral loads at 24 and 48 h p.i., (Figure 4(H)). The reduced loads of ZIKV RNA and E-protein at 96 h p.i., corresponds to the severe neuronal loss at this time point (Figure 4(G,H)). Detection of HSP70 in neuronal cell-derived exosomes further supported the presence of exosomal marker in both uninfected and infected exosomal lysates (Figure 4(H)). Furthermore, increased ZIKV RNA and E-protein loads were noted in neuronal exosomes with an increase in viral doses from 1, 2.5 and 5 MOI at 72 h p.i., (Figure 4(I,J)). Total protein profiles served as loading controls in the immunoblotting analysis (Figure 4(H,J)). Densitometry analysis from total exosomal lysates showed the quantitative differences in E protein (from both the time point and dose–response analysis) and HSP70 levels (time points) observed between the ZIKV infected (MOI 5) and uninfected controls (Supplementary Fig. 3D–F). These data suggest that cortical neuronal cell-derived exosomes contained ZIKV RNA and E-protein with enhanced loads.

### ZIKV RNA and proteins are infectious and are securely contained inside the exosomes for transmission

**Figure 5.**
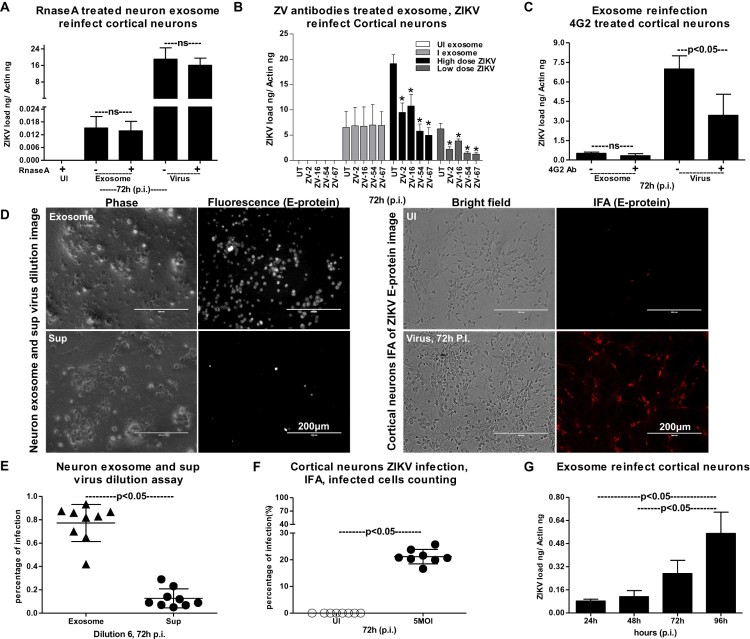
Exosomes from primary cultures of cortical neurons are infectious and transmit ZIKV to naïve neuronal cells. (A) Infections via freshly prepared exosomes from ZIKV-infected (MOI 5, 72 h p.i.) cortical neuronal cells or laboratory ZIKV viral stocks treated with RNaseA on naïve cortical neuronal cells (72 h p.i.) are shown. Untreated ZIKV-infected or RNaseA-treated uninfected exosome or untreated-virus stock groups serve as controls. (B) ZIKV loads (72 h p.i.) in neuronal cells incubated with ZIKV-infectious exosomes or laboratory viral stocks (8 MOI as high dose or 0.8 MOI as low dose) treated with ZV-2, ZV-16 or ZV-54, ZV-67 antibodies is shown. Cells incubated with uninfected cell-derived exosomes serves as control for infectious exosome group. The untreated (no antibody treatment) groups included in all panels, respectively serves as controls. C) QRT-PCR analysis showing viral loads in cortical neurons treated with 4G2 antibody followed by infection with exosomes (collected from independent batch of ZIKV-infected (MOI 5; 72 h p.i.) cortical neurons) or laboratory viral stocks. Untreated groups (–) serve as controls. ns indicates no significance in exosome or virus-treated groups. (D) Representative fluorescent images showing detection of E-protein in neuronal cells infected via exosomes (upper panel) derived from independent batch of ZIKV-infected (MOI 5; 72 h p.i.) cortical neurons or infected using ZIKV laboratory viral stocks (for 72 h p.i., lower panel). Neuronal cells treated with exosome-depleted supernatant (sup, in a similar ratio) served as control for exosome group. Uninfected cells serve as control for ZIKV laboratory stock infected group. Scale bar indicates 200 µm in all panels. (E) Quantitative assessment of number of ZIKV-infected (positive for fluorescence, detected by 4G2 antibody) (MOI 5; 72 h p.i.) neuronal cells treated with exosomes (in dilution 6) or supernatant fractions is shown. (F) Quantitative assessment of number of fluorescent neuronal cells, (detected by 4G2 antibody) infected (MOI 5; 72 h p.i.) with laboratory ZIKV viral stocks is shown. G) ZIKV loads in cortical neuronal cells (at different time points of 24, 48, 72 and 96 h p.i., and 5 MOI) infected via infectious exosomes is shown in naïve cortical neuronal cells for transmission and replication of viral RNA. In panels A, B, and F, ZIKV loads are indicated based on NS5 transcripts normalized to mouse beta-actin transcript levels. *P* value determined by Student’s two-tail *t*-test is shown.

We performed RNaseA-treatment assays, to test the possibility that ZIKV RNA is not perhaps outside the exosomes (in PBS suspensions) and hence taken up by the recipient cells. Neuronal exosomes derived from ZIKV-infected cells (from 72 h p.i., 5 MOI) were freshly isolated (resuspended in PBS) and treated with RNase A (5 μg/ml, for 15 min, at 37°C). We did not find any differences in ZIKV NS5 transcript loads from infected-treated or untreated groups (Figure 5(A)). The uninfected group treated with RNaseA served as an internal control (Figure 5(A)). The laboratory generated viral stocks (5 MOI) with known titres (collected from 14 days p.i., Vero cell culture supernatants) were treated with RNaseA, in order to not exclude the effect of RNaseA on viruses. No differences were noted in viral loads determined from naïve cortical neuronal cells upon incubation with laboratory viral stocks prepared from RNaseA-treated or untreated groups (Figure 5(A)). Similar to our previous results with DENV2 [47], we assume that the flavivirus RNA genome is inside exosomes/virions and is not available for RNaseA-mediated degradation. Next, we tested whether exosome-mediated viral transmission is dependent on ZIKV E-protein in naïve cortical neuronal cells. We treated infectious exosomes (containing viral RNA and proteins inside exosomes) or laboratory prepared viral stocks (high infectious dose of MOI 8 or low dose of MOI 0.8) with antibodies (3 µg of each) ZV-2, ZV-16 or with highly potent neutralizing antibodies ZV-54, ZV-67 [48] for 4 h at 37°C followed by infection (for 72 h) of naïve mouse cortical neuronal cells. No differences in ZIKV loads were noted in neuronal cells incubated with infectious exosomes that were treated with either ZV-2 or ZV-16 or with ZV-54 or ZV-67 neutralizing antibodies in comparison to their respective untreated controls (Figure 5(B)). Antibody treatments of exosomes from uninfected cells were used as internal controls for the infectious exosome group (Figure 5(B)). We found a significant reduction in ZIKV loads in neuronal cells incubated with ZV-2, ZV-16 or ZV-54, ZV-67 antibodies treated with high (8 MOI) or low (0.8 MOI) doses of laboratory viral stocks when compared with their respective untreated controls (Figure 5(B)). Also, ZV-54 and ZV-67 antibodies treatments with high (8 MOI) or low (0.8 MOI) doses of laboratory viral stocks showed significantly (*P* < 0.05) higher neutralization effects when compared with ZV-2 or ZV-16 antibodies, in both the high and low viral doses groups (Figure 5(B)). Additionally, we performed immunofluorescence assays (IFA) on neuronal cells (using 4G2 monoclonal antibody for detection). Similar results were obtained from infectivity assays showing no differences in ZIKV E-protein staining of neuronal cells that were incubated with infectious exosomes that were either kept untreated or treated with ZV-2, ZV-16 or highly potent ZV-54 or ZV-67 neutralizing antibodies (Supplementary Fig. 4). In case of neuronal cells incubated with high (8 MOI) or low (0.8 MOI) viral doses of ZIKV (pre-treated with either ZV-2, ZV-16 or ZV-54, ZV-67 antibodies), neutralization effects corresponding to lower detection of viral E-protein/staining and less fluorescently labelled cells was evident in comparison to their respective untreated controls (Supplementary Figs. 5 and 6). It was also noted that in comparison to ZV-2 and ZV-16, the highly potent ZV-54 or ZV-67 antibodies had stronger neutralization effects with dramatically reduced E-protein stained positive cells. Next, we treated cortical neurons with 5 µg of 4G2 antibody (that poorly neutralizes ZIKV), followed by infection via exosomes isolated from ZIKV-infected (72 h p.i., 5 MOI) neuronal cells, to analyse if treatment with 4G2 antibody affects or blocks viral transmission (Figure 5(C)). No differences in viral loads were found in antibody-treated or untreated groups of neuronal cells upon incubation of infectious exosomes isolated from ZIKV-infected neurons (Figure 5(C)). Addition of ZIKV laboratory stocks with known titres (MOI 5) showed reduced viral loads in cortical neuronal cells treated with 4G2 antibody in comparison to the untreated controls (Figure 5(C)). These results suggest that E-protein is securely contained inside the exosomes. However, E-protein on the surface of the virions is accessible to ZV-2, ZV-16, ZV-54 or ZV-67 or 4G2 antibodies and treatment with these antibodies showed differential neutralizing effects upon use of laboratory viral stocks. These data further suggest that E-protein is perhaps not on the surface of exosomes and may not be required for mediating viral RNA and protein transmission via exosomes. To determine, if exosomes-mediated viral transmission is clathrin-dependent, we treated cortical neuronal cells with clathrin specific inhibitor (Pitstop-2; 30 µM for 15 min), and infected these Pitstop-2 treated cells with infectious exosomes derived from ZIKV-infected (5 MOI; 72 h p.i.) cortical neuronal cells. We did not find any differences in ZIKV loads (at 72 h p.i. of naïve cortical neuronal cells) in Pitstop-2 treated group in comparison to the DMSO (vehicle)-treated controls (Supplementary Fig. 7). These results suggest that exosome-mediated ZIKV transmission to naïve neuronal cells is clathrin independent.

To test whether exosomal RNA was infectious to naïve cortical neuronal cells, we determined the viral titres in exosomes by performing a virus dilution assay. Representative images from neuronal cells infected via incubations with exosomes or exosome-depleted supernatants (collected during the exosome isolation methods) are shown from the virus dilution assay (Figure 5(D)). Increased fluorescent signal (as determined by immunostaining with 4G2 antibody) was evident in neurons infected with infectious exosomes in comparison to infection via supernatant fraction (Figure 5(D)). As an internal control, we also performed the viral dilution assay on cortical neuronal cells by infecting with ZIKV laboratory virus stocks (5 MOI, 72 h p.i.). Exosome-mediated ZIKV-infected cells detected enhanced E-protein staining with 4G2 antibody in comparison to the supernatant-fraction-treated cells (Figure 5(D)). Phase contrast images (from both groups either treated with exosomes or laboratory viral stocks) showed similar morphology of infected-neuronal cells (Figure 5(D)). Quantification of fluorescent signals (determined by counting the percentages of fluorescently labelled infected cells staining the E-protein) in neuronal cells infected with exosome fraction in comparison to the supernatant fraction (for 72 h p.i.) further supported the microscopic observation (Figure 5(E)). Dilution six was considered for both microscopic and quantitative analysis of infected cells from each replicate. The quantification of fluorescent signals (determined by counting the percentages of fluorescently labelled infected cells showing the E-protein staining by 4G2 monoclonal antibody) in neuronal cells infected with laboratory virus stocks of ZIKV (5 MOI, 72 h p.i.) showed infectivity of the viral stocks (Figure 5(F)). Next, we tested whether exosomes isolated from ZIKV-infected neuronal cells (from 72 h p.i., 5 MOI) are infectious and replicative in uninfected/naïve cells. QRT-PCR analysis showed that murine cortical neuronal cells treated with ZIKV-infected neuronal-cell-derived exosomes (isolated from an independent batch of infected cells) readily transmitted infectious exosomal RNA to uninfected recipient neuronal cells (Figure 5(G)). Also, we noted that infectious exosomal RNA replicated in the naïve/uninfected neuronal cells in a time-dependent manner at the tested time points of 24, 48, 72 and 96 h p.i. (Figure 5(G)). These data suggest that neuronal exosomal RNA is infectious, viable, and replicative in naïve recipient cortical neuronal cells of the CNS.

### Neutral Sphingomyelinase SMPD3 (nSMase2) facilitates ZIKV infection and transmission via exosomes

**Figure 6.**
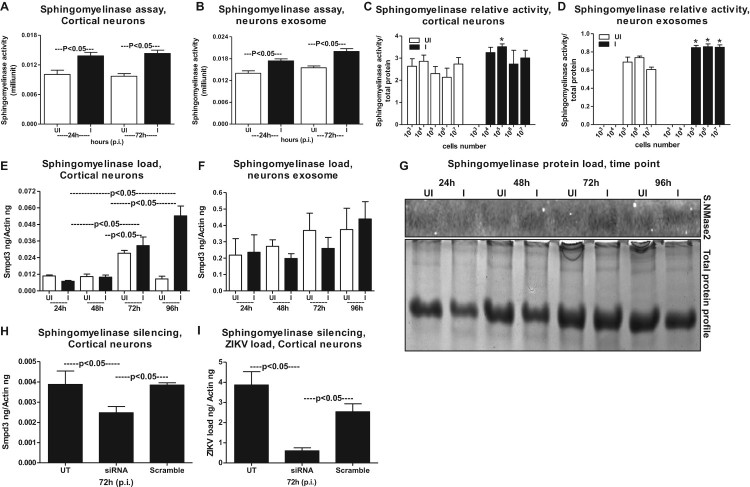
ZIKV induces expression of SMPD3, a neutral sphingomyelinase critical for exosome production and release. Neutral sphingomyelinase activity assays performed on cell lysates (A) or exosomal lysates (B) are shown. Neuronal cell and exosomal lysates derived from ZIKV-infected (MOI 5) was used from 24 and 72 h p.i. Lysates from uninfected neurons or exosomes served as controls. The *Y*-axis represents activity of neutral sphingomyelinases (shown in milliunits) and *X*-axis indicates the hours in each panel. SMPD3 activity (measured over the amount of total proteins) in neuronal cells (C) or in neuronal cell-derived exosomes (D) determined from different number/densities of plated cells is shown. White bar indicates uninfected and black bar denotes infected groups. Asterisk indicates significance. (E) QRT-PCR analysis showing expression of *smpd3* mRNA transcripts in uninfected (UI) or ZIKV-infected (I) (MOI 5) cortical neuronal cells or in cortical neuronal cell-derived exosomes (F) isolated at different time points (24, 48 72 and 96 h p.i.). (G) Immunoblotting analysis showing SMPD3 protein levels in uninfected (UI) or ZIKV-infected (I) cortical neuronal cells at 24, 48 72 and 96 h p.i.. Total protein profile image from coomassie-stained gel served as control. QRT-PCR analysis showing expression of *smpd3* (H) or ZIKV NS5 mRNA transcript loads (I), to reveal silencing efficiency (H) or viral burden (I) in untreated (UT) or *smpd3* specific siRNA or scrambled siRNA-treated ZIKV-infected (MOI 5; 72 h p.i.) murine cortical neuronal cells. Untreated cells were used as controls. The *smpd3* or ZIKV NS5 gene transcript levels were normalized to mouse beta-actin levels. *P* value determined by Student’s two-tail *t*-test is shown.

It has been shown that the membrane-associated enzyme SMPD3 (nSMase2; 71 kDa) is one of the most studied neutral Sphingomyelinase that is activated by anionic phospholipids such as phosphatidylserine (PS) and phosphatidic acid (PA) [38–41]. Activation of SMPD3 in cells plays an important role in the cellular responses [38–41]. We tested the activation of nSMase upon ZIKV infection in both neuronal cells and exosomes re-suspended in PBS. At both tested time points (24 and 72 h p.i.), neutral Sphingomyelinase activity (measured as milliunits) was significantly (*P* < 0.05) enhanced in ZIKV-infected cortical neurons and in exosomes derived from these neuronal cells (Figure 6(A,B)). In order to address if the increase in Sphingomyelinase activity is dependent on the cell number and production/release of exosomes, we plated murine cortical neurons at different cell densities and collected cells and exosomes at 72 h p.i., from ZIKV infected (5 MOI) or uninfected controls. Not many differences were found in Sphingomyelinase activity from cells plated at different densities, perhaps the growing cells had maintained the same fold of SMPD3 activity (Figure 6(C)). We found an increase in Sphingomyelinase activity in exosomes derived from varying number of cells plated in increasing densities and this also reflected an increase in exosome release from ZIKV infected cells (Figure 6(D)). This data also correlate with the increasing amounts of total proteins extracted from either cells or exosomes collected from ZIKV-infected (5 MOI) or uninfected controls (Supplementary Fig. 8A–F). No significant (*P* < 0.05) differences were noted in total protein amounts collected from ZIKV-infected or uninfected cells and neither from total proteins estimated from ZIKV-infected or uninfected exosomal lysates (Supplementary Fig. 8(B,C, E,F)). We also found that ZIKV infection, significantly (*P* < 0.05) upregulated *smpd3* transcripts levels in neuronal cells at both 72 and 96 h p.i. (Figure 6(E)). No differences in *smpd3* transcript levels were noted in exosomal fractions collected from ZIKV-infected group, when compared with the uninfected controls (Figure 6(F)). Since exosomal *smpd3* transcripts were normalized to actin (Figure 6(F)), we double confirmed that this is not due to lower actin transcripts in exosomes. QRT-PCR analysis for GAPDH loads and normalization to the *smpd3* transcripts with either of the housekeeping genes showed no significant (*P* < 0.05) differences (Supplementary Fig. 8G). Similar to the *smpd3* transcripts levels, the SMPD3 protein loads were upregulated at both 72 and 96 h p.i., in ZIKV-infected neuronal cell lysates in comparison to the uninfected controls (Figure 6(G)). Total protein profile gel image served as loading control (Figure 6(G)). Densitometry analysis from total cell lysates showed the quantitative differences in SMPD3 protein levels (from different time points) observed between the ZIKV infected (MOI 5) and uninfected controls (Supplementary Fig. 8H). Silencing of *smpd3* by siRNA treatment showed significantly (*P* < 0.05) lower loads of *smpd3* transcript levels at 72 h ZIKV p.i., in comparison to the untreated or scrambled siRNA-treated control groups (Figure 6(H)). Significantly (*P* < 0.05) reduced ZIKV loads were noted in *smpd3*-siRNA- treated cells in comparison to untreated or scrambled-siRNA-treated control groups (Figure 6(I)). These data suggest that SMPD3 facilitates ZIKV infection in murine cortical neurons.

### Exosome-release inhibitor GW4869 reduced ZIKV loads and transmission through SMPD3

**Figure 7.**
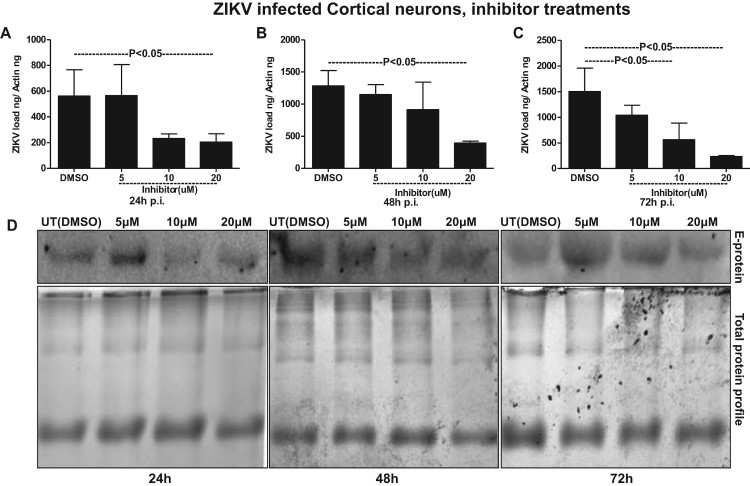
Treatment with exosome inhibitor affects ZIKV burden in murine cortical neuronal cells. QRT-PCR analysis showing expression of NS5 mRNA transcripts to reveal viral burden in cortical neuronal cells infected with ZIKV (MOI 5) at different time points of 24 (A), 48 (B) and 72 (C) h p.i. ZIKV NS5 gene transcript levels were normalized to mouse beta-actin levels. *P* value determined by Student’s two-tail *t*-test is shown. (D) Immunoblotting analysis showing levels of viral E-protein in ZIKV-infected (MOI 5) cortical neuronal cell lysates from different time points (24, 48 and 72 h p.i.) of infection. GW4869 inhibitor was treated for four hours at tested doses of 5, 10 or 20 µM and at indicated time points of 24, 48 and 72 h p.i.. Lysates prepared from DMSO-treated cells serves as mock control. Total protein profile images from coomassie-stained gels served as control in (D).

**Figure 8.**
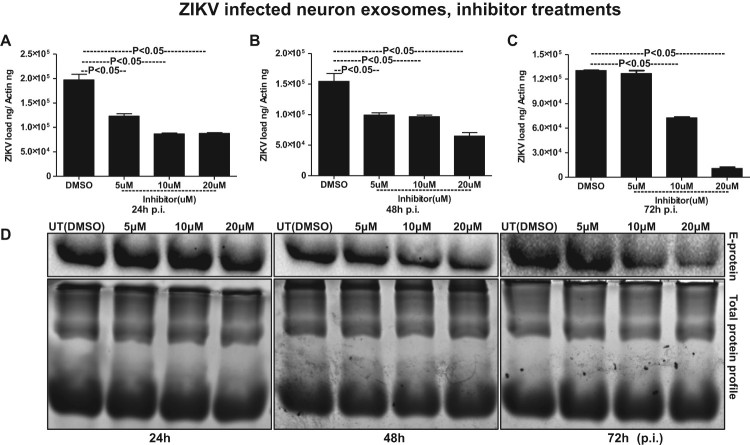
Treatment with GW4869 affects ZIKV burden in murine cortical neuronal cell-derived exosomes. QRT-PCR analysis showing expression of NS5 transcripts to reveal viral burden in ZIKV-infected (MOI 5) cortical neuronal cell-derived exosomes collected at different time points of 24 (A), 48 (B) and 72 (C) h p.i.. ZIKV NS5 gene transcript levels were normalized to mouse beta-actin levels. *P* value determined by Student’s two-tail *t*-test is shown. (D) Immunoblotting analysis showing levels of viral E-protein in ZIKV-infected (MOI 5) cortical neuronal cell-derived exosomal lysates at different time points (24, 48, and 72 h p.i.) of infection. GW4869 inhibitor was tested at 5, 10, or 20 µM concentrations and at indicated time points. Lysates prepared from DMSO-treated cells serves as mock control. Total protein profile images from coomassie-stained gels served as control in (D).

It has been reported that inhibition of nSMases with inhibitor GW4869 or by silencing through siRNA alters the metabolite composition of cells and extracellular vesicles (EVs) thereby, blocking the release of exosomes [18,39,49]. We tested the effects of GW4869 inhibitor on exosome budding and release upon ZIKV infection in murine cortical neurons. GW4869 inhibitor treatment (20 µM) showed significantly (*P* < 0.05) reduced loads of ZIKV in cortical neuronal cells infected (5 MOI, and at different time points of 24, 48 and 72 h p.i.) (Figure 7A–C). No differences in ZIKV loads were noted when neuronal cells were treated with lower doses (5 and 10 µM) of GW4869 inhibitor. Also, it was noted that the reduction in ZIKV loads in murine cortical neurons was dose- and time-dependent, where at 72 h p.i. GW4869 treatments at 20 µM concentrations showed a dramatic reduction in viral loads when compared with the mock (vehicle; DMSO) treated groups (Figure 7A–C). Immunoblotting with 4G2 antibody showed reduced ZIKV E-protein loads upon GW4869 treatment at 20-µM concentrations in comparison to the other tested doses of 5 and 10 µM and respective time points (24, 48 and 72 h p.i.,) and DMSO controls (Figure 7(D)). At 72 h p.i., treatment with GW4869 at 20 µM concentration, showed greater reduction in ZIKV E-protein loads in comparison to the early tested time points of 24 and 48 h p.i., (Figure 7(D)). Total protein profile gel images from respective time points (24, 48 and 72 h p.i.) of ZIKV-infected neuronal cells served as loading control (Figure 7(D)). In addition to the murine cortical neuronal cells, we analysed the potential effects of GW4869 inhibitor on exosome release from ZIKV-infected cortical neurons. QRT-PCR analysis showed that exosomes derived from cortical neuronal cells treated with GW4869 inhibitor (at all tested doses of 5, 10 and 20 µM; and time points of 24, 48 and 72 h p.i.) had significantly (*P* < 0.05) reduced loads of ZIKV RNA in comparison to the DMSO control (Figure 8(A–C)). This reduction in ZIKV loads was more evident in exosomes, when compared with the reduction observed in cortical neuronal cells that released these exosomes (Figure 7(A–C) and Figure 8(A–C)). Similar to reduced ZIKV RNA, we found that ZIKV E-protein loads were also considerably reduced in cortical neuronal cell-derived exosomes at all the tested doses (5, 10, and 20 µM) and time points (24, 48 and 72 h p.i.) in comparison to their respective DMSO controls (Figure 8(D)). Total protein profile gel images from different time points (24, 48 and 72 h p.i.) of ZIKV-infected neuronal cell-derived exosomes served as loading control (Figure 8(D)). Densitometry analysis from total cell (for data shown in Figure 7(D)) and exosomal (for data shown in Figure 8(D)) lysates revealed the quantitative differences in E protein loads (from the GW4869 time points and dose–response immunoblot analysis) observed between the ZIKV infected (MOI 5) and uninfected controls (Supplementary Fig. 9A–C; for immunoblots shown in Fig. 7D and Supplementary Fig. 9D–F; for immunoblots shown in Fig. 8D). Furthermore, we found that upon treatment with GW4869 inhibitor, *smpd3* transcript levels in both cortical neurons (Supplementary Fig. 10A–C) and neuronal exosomes (Supplementary Fig. 10D–F) were significantly (*P* < 0.05) reduced (at 20 µM dose in cortical neurons and all tested doses of 5, 10 and 20 µM in neuronal exosomes at time points of 24, 48 and 72 h p.i.) in comparison to their respective DMSO-treated controls (Supplementary Fig. 11). Similar to ZIKV loads, *smpd3* transcript levels were also lower in exosomes when compared with the infected-cortical neuronal cells (Supplementary Fig. 10). These data suggest that exosome release inhibitor GW4869, blocks SMPD3-mediated ZIKV transmission and infection in cortical neurons.

## Discussion

Neurotransmission also called synaptic transmission is a process by which neurons communicate with one-another and send electrical impulses and signalling molecules/chemicals including neurotransmitters [50]. Axonal terminals of the sending (presynaptic) neuron releases the neurotransmitters including other cargo that binds to and activates the receptors or channels on the dendrites of a receiving (postsynaptic) neuron. With this tightly regulated process of neurotransmission from sending neurons to the receiving neuron, the discharge of exosomes from sending neuronal cells and entry of exosomes into neighbouring receiving neuronal cells takes place on a continuous basis. Within a neural presynaptic terminal, two kinds of small membrane nanovesicles such as synaptic vesicles (SVs; that are ∼35–55 nm in diameter) and exosomes (inside Multivesicular Bodies (MVBs); that are 50–200 nm in diameter) are discharged [33,44]. Exosomes have been shown to represent a variety of intercellular exchange of effector molecules in order to allow presynaptic neurons to modify gene and protein expression in postsynaptic neurons [33,44]. Neuronal exosomes have shown to transfer both membrane and cytoplasmic proteins, functional lipids (involved in signal transduction) or RNA [33,44]. In receiving neuronal cells, exosomal mRNA will be translated and the small RNA, including the miRNA that mediates gene silencing is activated to bind specific genes for suppression [33,44]. Recent discoveries of functional RNA, miRNA and proteins in the exosomes has increased the attention that has led to the emergence of numerous studies in identifying novel molecules present in neuronal exosomes [18,19,29–32]. Due to the occurrence of RNA in the exosomes, we hypothesized whether exosomes are also carriers of viral RNA from the latest emerging ZIKV. Exosomes have been shown as vehicles of transmission for a variety of microorganisms, and our recent findings of Langat virus (LGTV; a member close to the tick-borne encephalitis virus; TBEV) and WNV transmission through neuronal exosomes has provided new insights into vector-borne flaviviral diseases drug discovery [18,32,51–57]. Our recent study has also shown that dengue virus (Serotype 2; DENV2) entire viral RNA genome is present and transmitted via mosquito cell-derived exosomes [47]. In this study, we have shown that ZIKV RNA and proteins are securely transported to the neuronal cells through infectious exosomes. So far, no studies have elucidated whether ZIKV-caused neurological manifestations such as microcephaly involves host neuronal exosomes.

Our discovery that neuronal cell-derived exosomes are the carriers of mosquito-borne flavivirus infectious RNA and proteins suggest a novel mode of ZIKV transmission. In our studies with ZIKV infections, the presence of MAP-2 (neuronal marker) and absence of GFAP (astrocyte glial marker) clearly indicated the complete neuronal differentiation of progenitor cells to cortical neurons in our studies with ZIKV infections. The data with weak or absence of MAP-2 staining, and positive DAPI staining showing enhanced ZIKV E-protein levels, suggested that perhaps there is neurite retraction due to induction of cell death in ZIKV-infected neurons. Increased neuronal death at 120 h p.i., in comparison to the 72 h p.i., of murine cortical neurons infected with ZIKV (MOI 5) suggests that longer incubation hours and increasing infectious dose could be responsible for the massive death of cortical neurons and their connections in developing brains. Also increased viral loads at 72 h p.i., (at both mRNA and protein levels) in neuronal cells and neuronal cell-derived exosomes, suggest a timing of peak induction in viral replication in neurons and their transmission through exosomes. The observation of high ZIKV loads in neurons suggests that viral RNA and proteins could be simply transferred in exosomes via axonal transportation. Early diagnosis and detection of ZIKV infections in mothers could possibly allow strategies to block the transport and/or dissemination of viral RNA and proteins that cause damage to the neonatal cells. We assume that cortical neurons are severely damaged leading to death and loss in networking that perhaps results in small brain size in microcephalic brains. It has been shown that endoplasmic reticulum stress in embryonic brains (*in vivo*) results in unfolded protein response during ZIKV infection and associated microcephalic condition [58]. Inductions in HSP70 loads during ZIKV-infection or in uninfected cells over the period of post incubations of cortical neuronal cells suggest an important role for this chaperone to protect cortical neurons from stress and toxic effects administered during longer post incubations and viral infections. Detection of enhanced HSP70 loads in neuronal exosomes may also suggest a similar neuroprotective function. Our multiple attempts with cryo-EM analysis revealed no detection of virions/viral particles in any collected images. We did not observe any virion(s) or viral particles neither inside nor outside exosomes. We have used 72 h p.i., as the selected time point for exosome isolation and cryo-imaging analysis. Usually for virus structure determination, concentrated supernatants collected from longer times of infection (7–14 days p.i.) with high viral titres ranging from 10^9^ to 10^12^ PFU/ml and high centrifugal forces around 200,000 g are reported [18,45,47]. These conditions were not used in our neuronal exosome preparations. In our analysis, exosomes were found to exist in heterogenous populations varying from sizes of 30–200 nm. However, larger exosomes or EVs were also observed in a size range of 200–350 nm. ZIKV-infected neuronal cell-derived exosomes showed higher percentage of smaller exosomes (0–200 nm in diameter) or larger EVs (200–500 nm in diameter) suggesting dense packaging or greater cargo transport through these exosomes, respectively. Presence of HSP70, along with CD63 and CD9 in fractions 3–5 suggested these fractions to be exosomes (perhaps containing exosomes 30–200 nm in size) due to the presence of enriched exosomal markers. Enhanced detection of ZIKV-E-protein in fraction four suggested that viral proteins are enriched in exosomes of sizes 100–200 nm that may correspond to fractions three and four. The presence of high amount of viral RNA and proteins in neuronal exosomes when compared with neuronal cells, suggest dense packaging of viral components in exosomes for transmission to neighbouring neurons. Infection of naïve recipient cortical neuronal cells with infectious exosomal fraction four containing enhanced viral E-protein showed the presence of more viral RNA and its correspondence to increased infectivity. Higher amounts of viral E-protein in exosomal fraction four directly correlated with increased transmission. Also, our data suggest that ample amount of viral RNA and proteins inside cells may randomly get transported into exosomes for transmission to neighbouring neurons resulting in dissemination within the brains. The observation of no differences in viral loads or infectivity via exosomes treated with RNaseA or antibodies (ZV-2, ZV-16 or ZV-54 or ZV-67), further suggests the presence of viral RNA and proteins inside exosomes. An antibody binding to the surface of the virus particles leads to neutralization of viral infectivity. The 4G2 monoclonal antibody has been shown as highly cross-reactive to several of the flaviviruses. However, a recent report has described 4G2 antibody as a poor neutralizing antibody for ZIKV [59]. Our study showed that the neutralization effects with 4G2 were lower when compared with the other tested antibodies such as ZV-2 or ZV-16. Furthermore, use of ZV-54 or ZV-67, the highly potential neutralizing antibodies, suggested ZV-2 or ZV-16 antibodies are less potential in comparison to the ZV-54 and ZV-67 antibodies. These findings are in agreement with the previous study that tested neutralization effects on different ZIKV strains [48]. However, similar results with no differences in viral loads or infection were observed with infectious exosomes upon use of any of these antibodies (ZV-2, ZV-16, ZV-54 or ZV-67 or 4G2), suggesting that viral E-protein is located inside the lumen of these vesicles and not exposed on the surface for binding to these neutralizing antibodies. Infections of naïve recipient cortical neuronal cells via infectious neuronal exosomes further suggest exosomes as novel modes for ZIKV transmission. We assume that exosome-mediated spread is very fast and efficient instead of matured virions-receptor mediated transmission. In exosomes, abundant amount of viral RNA and proteins can be transmitted and they can highly be infectious in recipient cells. Also, it is safe for the viral RNA and proteins to be protected and transported as cargo to recipient cells via secured exosomes that can avoid any immune checkpoints during transfer via longer routes such as periphery to the brain in vertebrate host.

Neutral Sphingomyelinase 2 (SMPD3/nSMase), or sphingomyelin phosphodiesterase is an enzyme that is responsible for cleaving sphingomyelin (SM) to phosphocholine and ceramide, a second messenger involved in multiple cellular responses including stress [38,39]. Our cryo-EM analysis indicated increased production and release of neuronal exosomes, upon ZIKV infection. We assume that increased activation and levels of SMPD3, in neuronal cells perhaps is associated with the increased release of exosomes upon ZIKV infection. Detection of SMPD3 mRNA levels showed no significant differences in neuronal exosomes, however, its higher enzymatic activity in both neuronal cells and exosomes suggest that SMPD3 is critical for the release of neuronal exosomes during ZIKV infection. We propose that similar to increased SMPD3 protein levels detected in neurons, exosomes perhaps have higher protein loads. Silencing of *smpd3* in cortical neurons showed a significant reduction in viral loads thereby, indicating a requirement for this neutral sphingomyelinase in mediating ZIKV infection. These data suggested that ZIKV-infection could enhance the production and/or release of exosomes from cortical neurons by increasing the protein and activity of SMPD3. The cell density independent increase in SMPD3 activity in exosomes in comparison to cells (from which exosomes were derived) suggests a strong role of this enzyme in production and release of exosomes. In cells, SMPD3 activity was mostly maintained as the growing cells retained same fold of activity that was independent of the cell numbers plated. In human macrophage THP-1 cells, SMPD3 has been shown to contribute to secretion of exosomes by triggering the budding of exosomes into MVBs [60,61]. Inhibition of SMPD3 either through shRNA or siRNA or GW4869 inhibitor treatment has been shown to inhibit the release of exosomes [49,54,61]. GW4869 is a pharmacological agent that affects neutral sphingomyelinase activity and blocks exosome production and release [49]. Very recently it has been shown that GW4869 can inhibit ZIKV infection by perhaps affecting the neutral Spingomyelinase-2 in human foetal astrocytes, however, no insights into the sphingomyelin metabolism was addressed [62]. GW4869 inhibitor is been also shown to inhibit the ceramide-mediated budding of MVBs and release of mature exosomes from MVBs [41,49,60,61,63]. The reduction in viral loads upon treatment with GW4869 (in a dose- and time-dependent manner) suggests that GW4869 may be considered as a candidate for the development of novel therapeutic to control ZIKV infection and transmission in and between cortical neuronal cells. In both cortical neurons and neuronal cell-derived exosomes, decreased ZIKV loads correlated with reduced SMPD3 activity and levels upon GW4869 treatment in a dose- and time-dependent manner. This data suggest that SMPD3 facilitates ZIKV infection and transmission through neuronal exosomes in order to disseminate the infectious viral RNA and proteins. It will be highly interesting to understand the mechanism of how SMPD3 facilitates ZIKV replication in neuronal cells. Our immediate future avenues will focus on this novel aspect to address the importance of SMPD3 and other neutral Sphingomyelinases in ZIKV infection and neuropathogenesis. The presence of higher ZIKV loads in exosomes when compared with neuronal cells suggests that ZIKV infectious RNA and proteins are perhaps abundantly been packaged and maintained to transmit through neuronal communication. We assume that higher amount of GW4869 inhibitor is essential in reducing the ZIKV loads in neuronal cells. We also noted that longer incubations and increased amounts of the inhibitor showed dramatic effects in reducing ZIKV loads in neuronal exosomes. The presence of higher loads of ZIKV infectious RNA and proteins in exosomes may lead to increased transmission causing neuropathogenesis and ultimately death/loss of cortical neurons. Our immediate future research efforts will address the underlying molecular mechanisms of SMPD3 and other neutral Sphingomyelinases in mediating the transmission of ZIKV and its detrimental effects in causing microcephaly in neonatal brains.

Given the importance of SMPD3 in mediating transmission of ZIKV through neuronal exosomes and GW4869 in inhibiting the viral loads in both neuronal cells and exosomes, we propose both as possible therapeutic candidates for controlling ZIKV infection and transmission in developing brains. Our long-term future avenues will also delineate the schemes that depend on neutral Sphingomyelinases including SMPD3, that lead to enhanced cortical neuronal death, and the underlying molecular mechanisms for ZIKV-caused microcephaly in developing/neonatal brains. The decreased size in microcephalic brains of ZIKV infected neonates is caused by a gradual decrease in neuron production or due to the rapid loss of dendritic connections [2,8,15,16]. Higher secretion and transport of infectious exosomes through axonal transport may transmit ZIKV infectious RNA and proteins to the dendritic branches of the receiving neurons, thereby decreasing the dendritic branching or arborization. Alternatively, if ZIKV uses exosomes for transmission in the CNS, it could spread the infectious viral RNA and proteins to neural stem cells and thereby manipulate the progenitor cells. Both these scenarios would ultimately lead to decreased neuronal numbers and act as a factor responsible for ZIKV-caused neuronal cell death and microcephaly. Delineating the importance of exosomes and exosomes-associated host molecules that mediate and facilitate ZIKV transmission would unravel the molecular mechanism(s) of infection by this neurotropic virus.

## Materials and methods

### Isolation and culture of murine cortical neurons and infection with ZIKV

C57BL/6 wild-type female mice with gestation period (day13) were purchased from Charles River Laboratories and allowed to reacclimatize in our animal facility. Following ethical rules and regulations in accordance with the institutional Animal Care and Use committee, we performed all mice experiments. All animal work in this study was carried out in strict accordance with the recommendations in the Guide for the Care and Use of Laboratory Animals of the National Institute of Health. Institutional Animal Care and Use Committee (Animal Welfare Assurance Number: A3172-01) approved protocol was used in this study (permit number: 16-017). Primary cultures of murine cortical neurons as disassociated cells were isolated from embryonic day- (E16) brains [18,64,65]. Murine cortical neuronal cells (2 × 10^5^) were seeded in a 12-well plate and cultures were established in complete neurobasal medium with 10% FBS. One day post-plating (DIV; Day *in vitro* 1), half of the medium was replaced with FBS-free media, to control the growth of glial cells. After 48 h of plating, we observed initiation of neuritogenesis, and cortical neurons were infected with ZIKV (MOI 5 or with different MOI 1, 2.5 or 5; in dose–response experiments) at 72 h post plating. Neurons were collected at either different time points (24, 48, 72, 96 or 120 h p.i., in infection kinetics and inhibitor treatment assays) or at 72 h p.i., in case of dose–response and other *in vitro* assays (as indicated in respective Figures and Figure legends). Following collection of neuronal cells and isolation of exosomes, we processed samples for RNA extractions and Real-Time Quantitative PCR (QRT-PCR). All culture medium and required supplements for neuronal culture isolation and maintenance were purchased from Invitrogen/ThermoScientific Inc. ZIKV PRVABC59 strain was obtained from BEI resources (catalog number: NR-50240) and propagated as per the instructions from the distributor. Due to increased viral loads in cortical neurons at 72 h p.i., we considered this time point for further analysis. Details for infection studies corresponding to the data shown in different figures is mentioned in the respective figure legends. Briefly, for re-infection experiments with infectious exosome fractions, 2 × 10^5^ neurons were co-incubated with 20 µl of neuronal cell-derived exosomal fractions. We used 400 µl (same ratio) of exosome-depleted supernatant fraction (collected from the step before PBS wash during exosome isolation, see our recent published study [18]) from neuronal cells processed for isolation of exosomes. Neuronal cells were harvested at different time points (24, 48, 72 and 96 h p.i.) and processed for RNA extractions followed by QRT-PCR.

### Immunofluorescence, Phase contrast and fluorescent microscopy

2–5 × 10^5^ neuronal cells were plated in complete neurobasal medium and allowed to adhere for overnight. Two days post-plating (DIV 2) neuritogenesis was observed, cortical neurons were infected with ZIKV (MOI 5; in time point samples or with different MOI’s 1, 2.5, 5 in dose–response experiment) at either 72 or 120 h post plating. Immunofluorescence (IFA) was performed as described [47]. Briefly, cortical neuronal cells were fixed with 4% PFA, permeabilized, blocked (3% BSA) and stained with MAP-2 antibody (Santa Cruz Biotechnology, Inc.) followed by detection with Alexa-Fluor 488 secondary antibody. Sequentially neurons were further stained for either GFAP antibody (Santa Cruz Biotechnology, Inc.) or 4G2 monoclonal antibody (that detects ZIKV E-protein) followed by detection with Alexa-Fluor 594 secondary antibody, respectively. Neuronal cell nuclei were detected with DAPI staining. Fluorescent images were obtained from 72 or 120 h p.i. cortical neuronal cells. Phase contrast images were collected from both uninfected and ZIKV-infected cells at different days or time points (24, 48, 72 and 96 h p.i.) or at 72 h p.i. in neurons infected with different MOI (infection with dose–response or virus dilution assays). For neutralization assays with ZV-2, ZV-16 or ZV-54, ZV-67 antibodies [48], neurons were fixed and processed as described above, and immunostained with 4G2 monoclonal antibody, followed by detection with Alexa Fluor 594 secondary antibody. Untreated cells represent no antibody treatments but were incubated either with infectious exosomes or with viral stocks (of known titres as described above). All cells were treated with DAPI to show cell nuclei in addition to bright field images that served as controls. Images were obtained using the EVOS Fluorescence System (Invitrogen/ThermoScientific Inc.) and 10X or 20X (in Figures 1, 5) or at 10X magnification (in Supplementary Fig. 1). Representative images are shown for each time point or group. Scale bar is shown on each representative image in the respective groups.

### MTT assays

MTT [3-(4,5-dimethythiazol-2-yl)-2,5-diphenyl-2H-tetrazolium bromide] assay was used to measure viability, which served as an index of living neuronal cells. Briefly, 5 mg/ml of stock solution was prepared by completely dissolving MTT (Sigma) in DMSO (100%) and followed by filter sterilizing (0.22 µm filter; Nalgene). Primary cultures of cortical neuronal cells were plated at the density of 1 × 10^4^ cells per well in a 96-well plate. The confluence of the cells was less than 100% in all wells. For infection, we infected neuronal cells with ZIKV (MOI = 5) and at different time points (24, 48, 72, 96 and 120 h) post infection, then we removed all medium from each well, and added 90 µl of fresh medium to each well. 100 µl of PBS was added to empty wells for background estimation. 10 µl of prepared MTT solution was added to all the wells including PBS containing wells that served as blank controls. Plates were covered in aluminium foils and incubated at 37 °C for 2–4 hrs (until purple precipitate was visible). 100 µl of DMSO solvent was added and plates were incubated at 37°C for another 15 min. Absorbance was read at 560 nm and for reference plates were read at 650 nm. The uninfected control maintained for 120 h post plating was considered to calculate the percentages of cell viability. Values from 650 nm reference read were subtracted from the values obtained at 560 nm to determine the neuronal cell viability numbers. Higher absorbance values indicated an increase in cell viability.

### RNA extraction, cDNA synthesis, and QRT-PCR analysis

Following manufacturer’s instructions, total RNA was extracted using Aurum Total RNA Mini kit (BioRad). RNA was extracted from cortical neurons or neuronal cell-derived exosomes infected (5 MOI) at indicated time points (24, 48, 72, 96 h p.i.) or with various doses (MOI 1, 2.5, 5) of ZIKV-infected group or uninfected controls. RNA was converted to cDNA using the BioRad iScript cDNA synthesis kit. The generated cDNA was used as template for the amplification and determination of viral burden. For detection of ZIKV replication, published forward and reverse primers for NS5 or E-gene region were used [66,67]. For SMPD3 gene expression analysis we used the following forward and reverse primers from published study [68]. To normalize the amount of templates, mouse *actin* amplicons were quantified with published primers [18]. Primers for beta-actin were used in parallel for QRT-PCR normalization. GAPDH primer sequences were designed based on the published study [69]. Equal amounts of mouse cDNA samples were used in parallel for beta actin, *gapdh* and ZIKV *E-gene* or *NS5* gene. The ratio of ZIKV *E-gene* or *NS5* amount/*beta actin* amount was used as an index to determine the rate of infection in each analysed sample. QRT-PCR was performed using iQ-SYBR Green Supermix and CFX96 instrument (BioRad, USA). Standard curves were prepared using 10-fold serial dilutions starting from standard 1–6 of known quantities of *actin* or *gapdh* or ZIKV *E-gene* or *NS5* gene fragments and QRT-PCR reactions were performed as described [18]. For RNaseA treatment, we isolated fresh exosomes from either uninfected or ZIKV-infected neuronal cells (2 × 10^7^), distributed the infected exosomes as treated (5 µg/ml RNase, 37°C for 15 min) or untreated groups. Uninfected exosomes (as similar volumes used in infected groups) treated with RNaseA were used as control. For ZIKV laboratory viral stocks (5 MOI), we treated the viral supernatants directly with RNaseA and incubated these samples on naïve primary cultures of murine cortical neurons. Non/poorly neutralizing antibodies ZV-2, ZV-16, or 4G2 (Clone D1-4G2-4-15) were obtained from BEI resources. The highly potent neutralizing antibodies ZV-54 and ZV-67 [48] were a kind gift from Dr. Michael S. Diamond’s laboratory, at the Washington University School of Medicine, Saint Louis, MO. For ZV-2, ZV-16 or ZV-54, ZV-67 or 4G2 antibodies treatments or neutralization studies, we treated either 3 µg (of ZV-2, ZV-16 or ZV-54, ZV-67) or 5 µg (of 4G2 antibody) of antibodies for 4 h at 37°C with either freshly isolated exosomes (20 µl of PBS suspension of exosomes from ZIKV-infected cells, 72 h p.i., 5 MOI) or with ZIKV laboratory viral stocks (8 MOI as high dose or 0.8 MOI as low dose for ZV-2, ZV-16 or ZV-54, ZV-67 antibodies or 5 MOI for 4G2 antibody treatments). In order to adjust the dose and reflect the infection of recipient cells with infectious exosomes, we have used either high or low doses of viral stocks with known titres for comparison. Exosomes were collected from independent batch of ZIKV-infected or uninfected cells and were used in this analysis. Neuronal cells were infected through infectious exosomes or viral stocks (pretreated with respective antibodies) for 72 h p.i., and collected for RNA extractions and processed for QRT-PCR. Untreated samples or neurons incubated with exosomes from uninfected cells served as experimental internal controls.

### Immunoblotting and densitometry analysis

Briefly, 2 × 10^7^ cortical neuronal cells were seeded on to six well-dishes for overnight incubation. Next day, we changed half of the complete media to no-FBS containing medium. Cells were infected with ZIKV (MOI 5) at 72 h post plating, and collected at different time points of 24, 48, 72, and 96 h p.i. or with different doses, of 1, 2.5 and 5 MOI. Cell culture supernatants were collected from the same cells at different time points (24, 48, 72, and 96 h p.i.) of infection or at 72 h p.i. (from different MOI samples) and processed for exosome isolation. Exosome fractions were collected after PBS wash (two times), and adherent cells were collected from the same plates (washed twice with 1 × PBS), and resuspended in modified RIPA buffer. Total protein amounts were estimated using BCA kit (Pierce/ThermoScientific, Inc.). Whole cell and exosomal lysates (10–30 µg) were separated on 12% SDS-PAGE gels. After gel electrophoresis, blots were blocked with 5% milk buffer and probed with either 4G2 (obtained from Millipore, Sigma or BEI resources) or HSP70 (Cell Signaling Technologies, Inc) or CD63 or CD9 monoclonal antibodies or SMPD3 polyclonal antibody (Santa Cruz Biotechnologies, Inc), followed by mouse or rabbit HRP-conjugated secondary antibodies (Santa Cruz Biotechnologies, Inc), respectively. Images showing total protein profiles obtained from Coomassie-stained gels served as loading controls. Antibody binding was detected with WesternBright ECL kit (Advansta, BioExpress). Blots were imaged using Chemidoc MP imaging system and processed using Image Lab software obtained from the manufacturer (BioRad). Densitometry analysis from total cell and exosomal lysates (from both the time point and dose–response immunoblot analysis) between the ZIKV infected (MOI 5) and uninfected controls was performed considering their respective total profile gel images and the respective band.

### Isolation of exosomes from cell culture supernatants

Exosomes were isolated by differential ultracentrifugation method [46]. Isolation procedure and modifications are shown in our recent study [18]. Briefly, 1–2 × 10^7^ cortical neuronal cells were seeded for exosome isolation in complete neurobasal medium (overnight). Neurons were infected with ZIKV (MOI 5) for 72 h p.i.. Briefly, cell culture supernatants were spun at 100,000 × g for 120 min. Supernatants collected after this spin served as exosome-depleted supernatant (EDS) fractions (used as control in our study). The pellet containing exosomes and any contaminants were washed with ice-cold PBS (another spin at 100,000 × g, for another 120 min). Resulting exosome pellet is referred as exosome fractions in our study. Freshly prepared exosome pellets were resuspended in PBS and either frozen at −80°C or used for subsequent evaluations and assays or were resuspended in RNA lysis (Biorad) or modified RIPA buffers (G-Biosciences, BioExpress) for total RNA or protein extractions.

### Cryo-Electron microscopy

We performed the cryo-EM as published in our recent study [18,47]. Briefly, purified concentrated suspensions of ZIKV-infected or uninfected exosomes resuspended in PBS were vitrified on carbon holey film grids and as previously described [70,71]. Frozen grids were stored under liquid Nitrogen and transferred to a cryo-specimen holder under liquid Nitrogen before loading into a JEOL 2200FS, or a JEOL 2100 electron microscopes (JEOL Ltd., 3-1-2 Musashino, Akishima, Tokyo 196-8558, Japan). Preliminary screening and imaging of exosomes was done using a 4k × 4k Gatan US4000 CCD camera and final imaging was done at indicated 40,000× magnification with a 5k × 4k Direct Electron Detector Camera using a low-dose imaging procedure. Images were acquired with a ca. 20 electrons/Å^2^ dose; the pixel size corresponded to 1.5 Å on the specimen scale. We used a 2.0–2.3 µm defocus range for imaging. For exosome size quantitations, we manually analysed the sizes using scale bar from cryo-EM images and counted exosomes of different sizes per image in each group. Three independent estimations and countings were performed without any bias. Percentages (for size determination) were calculated based on the total number of exosomes in each size range.

### OptiPrep™ density gradient exosome (DG-Exo) isolation

2 × 10^7^ cortical neuronal cells were infected with ZIKV (5 MOI and for 72 h p.i.). Uninfected neurons were used as controls. The detailed protocol for isolation of exosomes on density gradient is shown in our recent published study [18,47]. Briefly, supernatants (10 ml) from uninfected/infected cortical neurons were collected and centrifuged at 4°C (480 × g followed by 2000 × g for 10 min each to remove cell debris and dead cells). Cell culture supernatants were concentrated to ∼2 ml using the Corning Spin-X UF concentrators or centrifugal filter device with a 5 k nominal molecular weight limit (NMWL) (VWR). Concentrated cultures were processed for OptiPrep™ (DG-Exos) isolation as described [18,45]. Six individual fractions were collected from uninfected or infected groups (from top to bottom) manually after 18 h spin at 100,000 × g (with increasing density of iodixanol, and smaller size vesicles on bottom fractions) and diluted with 5 ml of sterile PBS. Fractions were centrifuged at 100,000 × g for 3 h at 4°C, followed by another PBS wash and spin at similar centrifugal forces. DG-Exo fractions were resuspended in 80 µl of PBS and stored at −80°C until further analysis. Exosomal fractions were either processed for Western blotting analysis to detect viral E-protein or the exosomal marker HSP70 or used in infection of naïve primary cultures of murine cortical neurons to determine the infectivity and transmission through infectious exosomal fractions.

### Viral dilution assay

We performed the virus dilution assay as described in [72]. Briefly, neuronal cells were seeded (at a density of 1 × 10^5^ cells/well in 225 µl of Neurobasal complete medium) on 96 well plates. Neurons were treated with either exosome fraction (20 µl) or with exosome-depleted supernatant fractions (EDS; 400 µl) or with laboratory ZIKV viral stocks (5 MOI) at 72 h post plating and incubated for additional 5 days. In each group, we had six different dilutions (1–6) and at least eight independent replicates in addition to the uninfected negative controls. Neurons were fixed with acetone-PBS mixture (3:1, for 20 min at −20°C) and plates were air dried, washed with 1 × PBS and blocked with 5% FBS-PBS-0.05% Sodium Azide for 15 min at RT. ZIKV E-protein was detected by incubation with 4G2 monoclonal antibody (overnight at 4°C), followed by three washes with PBS. Samples were incubated with Alexa-594 labelled mouse secondary antibody for 1 h at RT, followed by washes (3×) with PBS. Plates were analysed using EVOS fluorescence system (Invitrogen/Thermoscientific, Inc.) and cells were scored for fluorescence or the presence or absence of infection in comparison to the infected positive controls (infected with laboratory prepared virus stocks) or uninfected negative controls. Representative images from dilutions 10^6^ are shown. Percentage of infected neurons is shown from the same dilution for exosome and supernatant fractions.

### Neutral Sphingomyelinase activity assay

For determining the activity of neutral Sphingomyelinases, we used the Colorimetric Sphingomyelinase Assay kit from SIGMA-Aldrich and followed the instructions from the manufacturer. Briefly, we plated 1 × 10^7^ cortical neuronal cells and three-day post-plating infected with ZIKV (MOI 5) for either 24 or 72 h p.i.. In an independent assay to test if increase in SMPD3 activity is dependent/independent on cell number and exosome production and/release, we plated mouse cortical neuronal cells at densities of 10^3^, 10^4^, 10^5^, 10^6^ and 10^7^ cells per well in replicates and measured the total protein amounts and SMPD3 activity. We measured SMPD3 activity by either considering the same amounts of total protein (estimated by BCA assays), where SMPD3 activity was calculated over the total protein amounts as shown in Figure 6(C,D). We also considered equal volume of samples for determining the SMPD3 activity (as shown in Supplementary Figs. 9A and 9D). The total concentration of proteins estimated from uninfected or ZIKV-infected neurons or exosomes derived from these neurons is shown in Supplementary Figs. 9B and 9C; for cells or 9E and 9F; for exosomes. At different time points post infection, cell culture supernatants were collected and processed for exosome isolation. Both cell and exosomal lysates were resuspended in 1 × PBS and were frozen at −80°C. We used 50 µl for each time point sample (uninfected or ZIKV-infected) as 6 replicates. Absorbance from assay samples was measured at 655 nm. Calculations were performed using the Zero blank sphingomyelinase standard that is considered as background blank. All readings were substracted from the background values. Standard curves were plotted using the standard values and the amount of active sphingomyelinase present in the samples was determined based on the standard curve.

### siRNA transfections and inhibitor studies

For siRNA transfections and silencing of *smpd3*, we plated 1 × 10^6^ of cortical neurons in complete neurobasal medium, allowed to adhere overnight, and changed to half of medium without FBS on next day. For silencing of *smpd3*, specific siRNA (from Santa Cruz Biotechnologies, Inc.) were purchased and transfections were performed as per the manufacturer instructions and protocols. Cells were infected with ZIKV (MOI 5, at 72 h post plating) and collected at 72 h p.i. followed by RNA extractions and cDNA synthesis. Silencing efficiency of *smpd3* was analysed by QRT-PCR. For inhibition of exosome release from neuronal cells, we used the selective inhibitor (GW4869) for Neutral Sphingomyelinase 2 (nSMase2 or SMPD3) (Santa Cruz Biotechnologies, Inc.) dissolved in DMSO. Cytotoxicity of the inhibitor was first analysed, neurons did not show any toxicity at tested doses (of 1–20 µM). For inhibitor studies, neurons (2 × 10^5^ for RNA and 1 × 10^7^ for protein extractions) were plated in complete neurobasal medium. Cortical neurons were treated with GW4869 inhibitor (5, 10 or 20 µM) for 4 h at 72 h post plating, followed by ZIKV infection (5 MOI) at indicated time points (24, 48 and 72 h p.i., for both RNA and protein extractions). Cells treated with similar volume (we considered volume used for 20-µM GW4869) of DMSO were considered as control groups. Viral loads were determined at all tested time points followed by QRT-PCR analysis. Both whole cells and exosomal lysates (30 µg) were processed for immunoblotting with 4G2 monoclonal antibody, followed by secondary antibody to detect ZIKV E-protein.

### Statistics

Using GraphPad Prism6 software and Microsoft Excel, we analysed the statistical significance of difference observed in data sets. Non-paired, two-tail Student *t*-test was used (for data to compare two means) for the entire analysis. Error bars represent mean (+SD) values, *P* values of <0.05 were considered significant in all analysis. Statistical test and *P* values are indicated for significance in all figures.
